# Preservation of red blood cell antigenicity in a new storage solution *in vitro*

**DOI:** 10.1080/07853890.2022.2157476

**Published:** 2022-12-15

**Authors:** Sheng-Hui Tang, Hsin-Chung Lin, Jin-Biou Chang, Yung-Shu Chan, Hui-Fei Tang, Feng-Yee Chang, Tzong-Shi Chiueh, Bing-Heng Yang

**Affiliations:** a Division of Clinical Pathology, Department of Pathology, Tri-Service General Hospital, Taipei, Taiwan; b Blood Bank, Department of Pathology, Tri-Service General Hospital, Taipei, Taiwan; c Division of Blood bank, Department of Laboratory Medicine, Mackay Memorial Hospital, Taipei, Taiwan; d Department of Clinical Pathology, Far Eastern Memorial Hospital, Taiwan; e Division of Infectious Diseases and Tropical Medicine, Department of Medicine, Tri-Service General Hospital, Taipei, Taiwan; f Department of Medicine, National Defense Medical Center, Taipei, Taiwan; g Department of Laboratory Medicine, Linkou Chang Gung Memorial Hospital, Taoyuan, Taiwan; h Graduate Institute of Medical Sciences, National Defense Medical Center, Taipei, Taiwan; i Trace Element Research Center, Tri-Service general Hospital, Taipei, Taiwan

**Keywords:** RBC antigenicity, haemolysis, modified RBC preservation (storage) solution, polyethylene glycol (PEG)

## Abstract

**Introduction:**

Red blood cell (RBC) storage solution is used for suspending and preserving RBCs for later use in *in vitro* immunohematology testing. Proper RBC preservation is crucial for obtaining accurate results in RBC phenotyping and pretransfusion antibody screening tests. Haemolysis or RBC antigen degradation during storage can result in inaccurate RBC phenotyping, thereby decreasing the sensitivity of pretransfusion antibody screening and identification assays. The conventional RBC storage solutions usually contain adenosine, adenine, and antibiotics. We designed an RBC storage solution and determined whether it could preserve RBC integrity for 70 days.

**Materials and Methods:**

The new storage solution has a different formula from that of the conventional solution—in particular, it is strengthened with polyethylene glycol (PEG). The extent of haemolysis and hemagglutination reactivity of the RBC antigen systems, Rh, Duffy, Kidd, Lewis, MNS, P1, and the rare antigen Mi^a^ (which has a low prevalence antigen in most parts of the world but a higher prevalence in Taiwan), in the new RBC storage solution was compared with that of the conventionally preserved RBC storage solution.

**Results:**

The RBCs preserved in the new solution for 70 days retained a similar haemolysis grade as those preserved in the control solution for 28 days. Although both solutions largely preserved RBC antigenicity, the decline in RBC hemagglutination scores in new solution often occurred later than that in the control solution in most antigen phenotyping assays, especially labile antigens such as D, P1, and M.

**Conclusion:**

The new solution reduces haemolysis more effectively and preserves antigenicity throughout the 70-day storage period. Moreover, Mi^a^ antigen is more stable in the experimental group.

## Introduction

During storage, red blood cells (RBCs) undergo a series of changes collectively known as ‘storage lesions,’ eventually leading to their haemolysis [1,2]. Haemolysis is a leading cause of diagnostic error in laboratory tests [3]. Therefore, an appropriate RBC storage solution is crucial for minimizing haemolysis.

The few studies addressing *in vitro* RBC preservation have used only antibiotics and antioxidants in Alsever’s solution [4]. One study employed antioxidant additives and measured RBC storage survival based on phosphatidylserine exposure and haemolysis. The results revealed that a mixture of natural antioxidants provided a slight, although significant, decrease in phosphatidylserine exposure. No significant decrease in haemolysis was noted in the high haematocrit group, and phosphatidylserine exposure was only effective in the low haematocrit group [4]. Several microorganisms can induce haemolysis [5,6]; therefore, antibiotics such as cycloheximide [7], penicillin, streptomycin [8], neomycin sulphate, and chloramphenicol are added to Alsever’s solution [9]. The addition of cycloheximide caused negligible antigen deterioration after 8 weeks of storage, but the study did not evaluate the extent of haemolysis [7].

The loss of blood group antigens during storage is of greater concern than haemolysis, especially in pretransfusion screening and alloantibody identification tests [10]. In accordance with the quality assurance requirements of blood banks, screening and identification of panel cells at least once a month are required because decreasing and lost antigenicities due to improper storage may reduce sensitivity and yield false-negative results.

The stability of blood group antigens varies widely after a long storage period in conventional solution, the Alsever’s solution [11]. The Lewis and P antigens are the most labile blood typing antigens [12,13], whereas the ABO and Rh antigens are generally considered stable. However, a 40% decrease in the D antigen was reported after 3 weeks of storage in Alsever’s solution [14].

Various blood typing systems exist, including Rh (C, D, E, c, e), Duffy (Fy^a^, Fy^b^), Kidd (Jk^a^, Jk^b^), Lewis (Le^a^, Le^b^), P (P1), MNS (M, N, S, s), and Mi^a^ antigens. Of them, Mi^a^ antigen has been reported to appear in RBCs with several Miltenberger phenotypes which belong to MNS blood group system. In particular, Mi^a^ has a low prevalence in most parts of the world but has a higher prevalence in Southeast Asian populations and in Taiwan. The highest Mi^a^-positive prevalence was recorded in Taiwan’s indigenous Ami population (88.4%), along with the second or third highest in relation to alloantibody incidence in Taiwan [15,16]; it is therefore included in the domestic screening cell panels for regular pretransfusion tests at blood banks. Despite its importance in transfusion practices in Taiwan, the endurability of the Mi^a^ antigen during storage remains under investigation.

An experimental RBC storage solution was recently developed by Metek Lab, Taipei, Taiwan. The solution’s unique formula includes polyethylene glycol (PEG) added to a modified Alsever’s solution to enhance its preservation ability. PEG is a polymer that is widely used in the biotechnology field as a cell fusion agent [17], mRNA vaccine stabilizer [18], cell membrane stabilizer [19], and potentiator of RBC antigen-antibody reactions [20]. It has been demonstrated to reduce hemolysis rates [21]. In this study, we evaluated the performance of the new solution for both preserving RBC antigenicity and delaying hemolysis during storage.

## Materials and methods

### Preservation solution

We compared a commercially available conventional RBC storage solution (K1150 red cell storage solution, Sanquin, Amsterdam, the Netherlands) as the control solution with the new RBC storage solution (Metek Lab, Taipei, Taiwan; storage duration: 70 days) as the experimental solution. The new storage solution has a different formula from that of the conventional solution—in particular, it is strengthened with PEG ((C_2_H_4_O)_n_H_2_O, product no. 202444, Sigma-Aldrich Chemie, USA). However, for the protection of trade secrets, the exact formula of the new solution cannot be disclosed.

### RBC cell panel and antibodies

The integrity of the Rh (C, D, E, c, e), Duffy (Fy^a^, Fy^b^), Kidd (Jk^a^, Jk^b^), Lewis (Le^a^, Le^b^), P (P1), MNS (M, N, S, s), and Mi^a^ antigens in the conventional and new solutions was periodically evaluated during the 70-day storage period. The cell panels used for evaluating the RBC antigen antibody reaction (including cell panel SI: C + D+E − c − e+, Fy(a + b−), Jk(a + b−), Le(a + b−), P1(+), M − N + S − s+, and Mi^a^(−); Cell Panel SII: C − D + E + c + e−, Fy(a − b+), Jk(a − b+), Le(a − b+), P1(+), M + N − S + s+, and Mi^a^ (−)) were acquired from Sanquin (Amsterdam, the Netherlands), and Mi^a^-positive cells (M + N − S + s+, Mi^a^(+)) were acquired from Formosa Biomedical Technology Corporation (Taipei, Taiwan). We utilized cell panel SI, SII as negative controls for most antigens, except for D, P1 and s. RBC antibodies, the anti-D, C, E, c, e, Fy^a^, Fy^b^, Jk^a^, Jk^b^, Le^a^, Le^b^, S, s, M, and P1 antibodies, were also acquired from Sanquin, and the Taiwan Blood Service Foundation (Taipei, Taiwan) provided the anti-Mi^a^ antibody. No false positive/non-specific antibody bindings were observed. The cells in each solution were maintained at 2–8 °C for the following experiments.

### Comparison testing of two storage solutions

The experimental and control solutions were compared in terms of the extent of haemolysis and hemagglutination testing (RBC antigen–antibody reaction) by using titre scores. For hemagglutination testing, the antibodies with titre scores and the cell panels with known RBC antigens were used. Both analyses were performed in the blood banks of two medical centres in north Taiwan (Mackay Memorial Hospital and Tri-service General Hospital). There was no significant difference of hemagglutination results (macroscopically interpretated) between the two medical centres.

### Haemolysis assay

The extent of haemolysis was measured using spectrophotometry, and the testing sample (3% of the cell suspension of the cells panels) was separately refrigerated in different vials (Sterilized fluorinated ethylene propylene 10-mL centrifuge tubes, Nalgene, Rochester, USA) for testing on days 12, 21, 28, 35, 42, 49, 56, 63, and 70 for determining the qualified cell preservation period. To calculate the shelf life of reagent RBCs, consideration of the time elapsed between the manufacturing date and experiment initiation date is crucial. Accordingly, we defined the manufacturing date as day 0, and the experiment was initiated on day 12. In other words, we received the Sanquin panel reagent RBCs on day 12 and then conducted experiment by transferring reagent RBCs to conventional and new solutions. Additionally, the two medical centres needed to coordinate to conduct the experiment simultaneously, and day 12 was the mutually available date for both centres. The limit of storage period was set at day 70, as we believed that would be sufficient compared with the expiration date of the current RBC solution. So far, only one study has reported that storage period of reagent RBCs beyond 70 days (13-week, approximal 90 days) after treatment with 0.025% glutaraldehyde. Although the calculation method for hemagglutination scores used in that study [22] is different from our study, the score points of all RBC group antigens remained the same even storage for more than 70 days. Accordingly, we deemed ending at day 70 for evaluating the new storage solution as suitable. In addition, the expiration duration of commercial RBC reagents is almost shorter than 70 days (Markropanel 16 (P) of Sanquin: 42 days; Screen Cell I/II/III of Formosa Biomedical Technology Corporation: 52 days; Reagent Red Cells of Ortho-Clinical Diagnostics: 63 days; Panoscreen I and II & Pancell-10 of Immucor, Inc.: 67 days). In each assay, each of the following four groups (SI cell with original preservation solution, SII cell with original preservation solution, SI cell with new preservation solution, SII cell with new preservation solution, respectively) was evaluated with six repeats per group (*n* = 216). For each testing sample, cell panels were first centrifuged for 15 s at 1000 × g using the KUBOTA KA-2200 centrifuge (Osaka, Japan) to separate the storage solution before testing. The degree of hemolysis was evaluated by measuring the absorbance of the solution at 540 nm by using a MultiSkan GO microplate reader (Thermo Fisher Scientific, Waltham, MA, USA).

### Hemagglutination testing with titre score

All antibodies were manually titrated to provide a score of 2+ or 1 + s for the hemagglutination experiments for each antibody. Because we evaluated the extent of loss of antigenicity during an extended storage period, we deemed starting at 2+ or 1 + s reaction as suitable. It appears that the 2+ or 1 + s reactions are relatively weaker, but strongly visible enough for interpretation. In addition, the extent of reaction decline would be more obvious than if compared with a higher baseline score. 50 μL of RBC and 100 μL of antisera were used for each test. Hemagglutination reactions with anti–C, D, E, c, and e antibodies were evaluated through immediate spinning (1000 × g for 15 s) and with anti–Fy^a^, Fy^b^, and s antibodies through an indirect antiglobulin test. The hemagglutination reactions of the other antigens (Jk^a^, Jk^b^, P1, M, N, and S) were measured after they were incubated individually with cell panels at room temperature (25 °C) for 15 min and at 4 °C for 15 min for Le^a^ or Le^b^. All hemagglutination results were recorded according to the Technical Manual of the American Association of Blood Banks (4+ = 12, 3+ = 10, 2+ = 8, 1+ = 5, w+ = 2, negative = 0). The triplicated hemagglutination scores of all blood units were tested on day 12, 21, 28, 35, 42, 49, 56, 63 and 70.

### Statistical analysis

Statistical analysis was conducted using Microsoft Excel (Microsoft, Redmond, WA, USA). Data were compared using the paired two-tailed Student’s *t*-test (for haemolysis) or chi-square test (for hemagglutination score). The results were determined using mean values and considered statistically significant at *p* ≤ 0.05.

## Results

### Significantly lower haemolysis with the experimental solution

**Figure 1.**
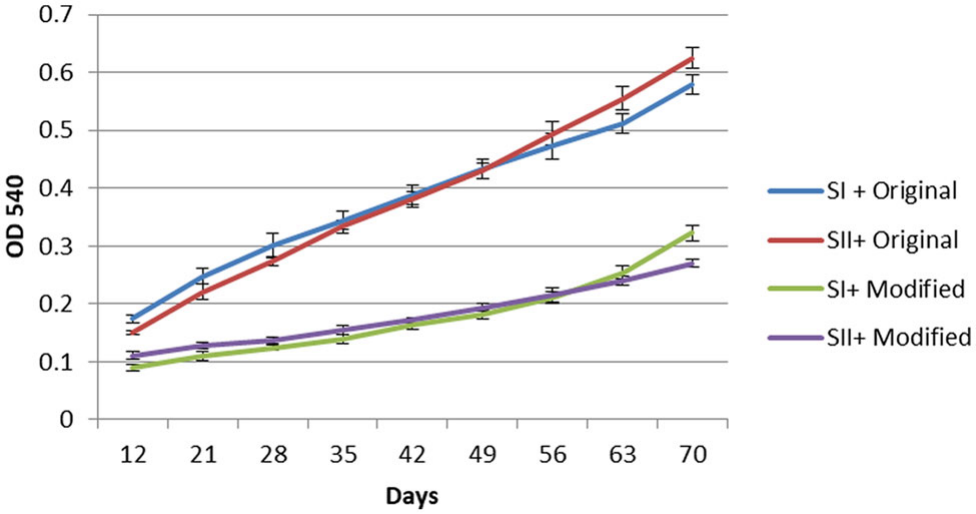
Hemolysis indexes for RBCs preserved in control and experimental solutions during storage. Depending on the combinations of the screen cell sets and preservation solutions (SI + control, SII + control, SI + experimental, SII + experimental [n = 54 each, 6 (triplicate in two medical centers) x 9(days) = 54]), the experimental solution provide superior protection against hemolysis than control solution. All the results shown in Figure 1 are means and error bars indicate standard deviation.

Significantly lower hemolysis indexes were obtained for the RBCs preserved in the experimental solution than in the control solution (Figure 1). Both cell panels presented significantly lower OD540 absorbance after day 12 of storage (*p* < 0.05).

The RBCs preserved in the experimental solution for up to 70 days exhibited the same haemolysis index (OD540 absorbance of approximately 0.3) as those in the control solution at 28 days (i.e. the indicated 28-day expiration).

### Lower but detectable antigens on cell panels throughout 70-day storage

**Figure 2.**
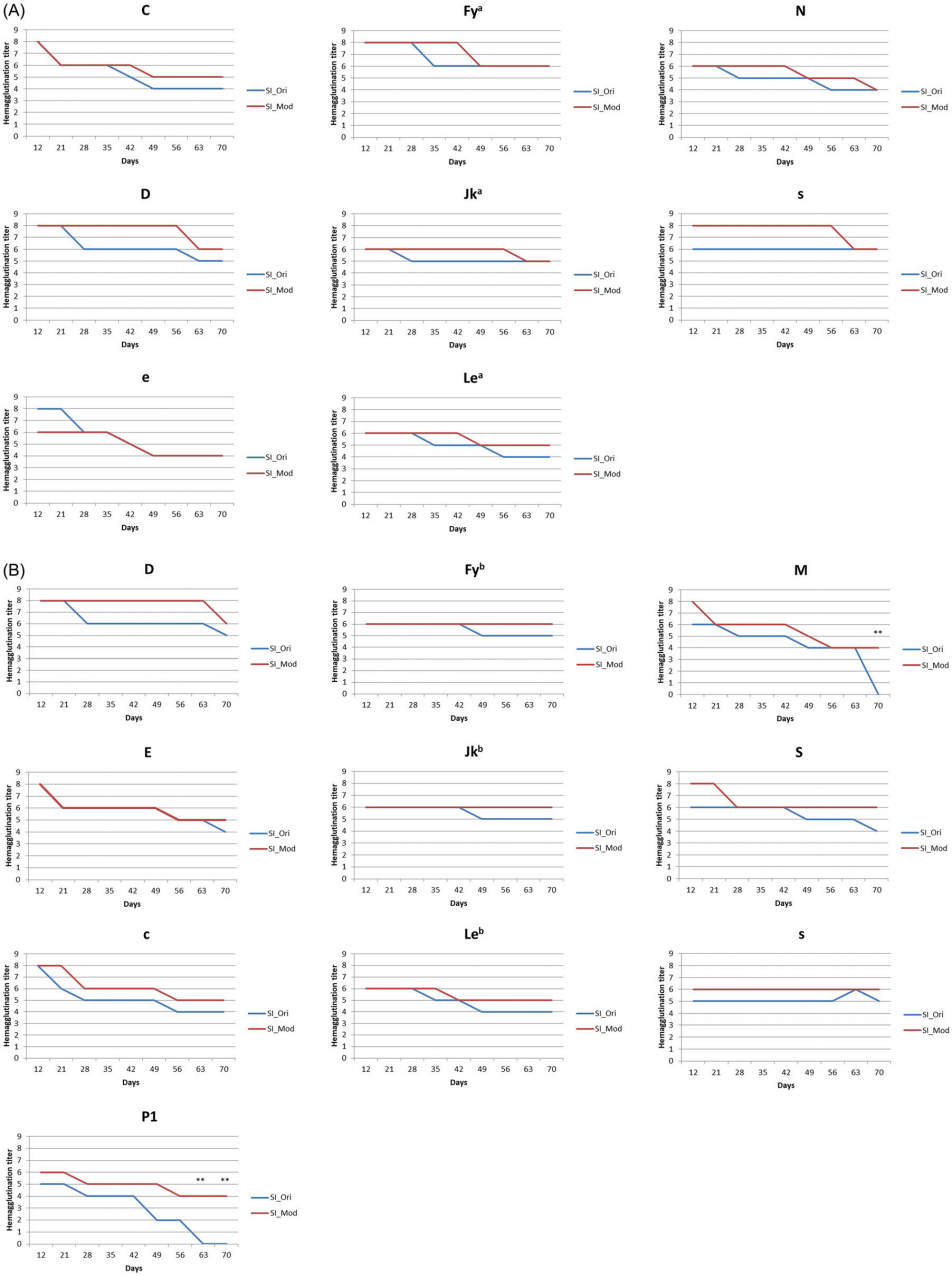
(A) SI screen cell antigenicity changes. SI_Ori: SI screen cells preserved in the control solution, SI_Mod: SI screen cells preserved in the experimental solution (the results were all quadruplicated which there were duplicate in two medical centers). SI cell antigenicity changed over time. All antigens remained detectable in both solutions, but the experimental RBC preservation solution had the same or higher agglutination scores during the experiment. The results presented in (A) are means, and error bars (standard variation) are not shown because the repeated results were the same. (B) SII screen cell antigenicity changes. SII_Ori: SII screen cells preserved in the control solution, SII_Mod: SII screen cells preserved in the experimental solution (the results were all quadruplicated which there were duplicate in two medical centers). According to the changes in SII screen cell antigenicity, M and P1 antigens were more prone to lost antigenicity. Compared with the control solution, the experimental solution effectively preserved these antigens. (**: *p* < 0.01). The results presented in Figure 2B are means, and error bars (standard variation) are not shown because the repeated results were the same.

All the antigens declined gradually in various grades for cell panel SI; however, they were still detectable at the end of the storage period (Figure 2(A)). Generally, a lower hemagglutination score of 1–4 was observed for every antigen on cell panel SI after the storage period despite preservation in the experimental solution. The hemagglutination scores of C and e declined by 4 points from day 12 to day 70 in the control group, but a smaller decline was observed in the experimental group.

The antigens on cell panel SII presented a similar 1–4 grade decline as the antigens on cell panel SI, but the cells had detectable hemagglutination scores for their specific monoclonal antibodies after the standard 28-day storage period in both storage solutions (Figure 2(B)). However, the M and P1 antigens were preserved in the experimental group, with hemagglutination reactivity of 50.0% and 66.7%, respectively, on day 70, whereas in the control group, no reactivity was observed on day 70. The hemagglutination scores of c and E declined by 4 points from day 12 to day 70 in the control group, but a smaller was observed in the experimental group. The hemagglutination scores of M and P1 were significantly higher in the experimental group (**: *p* < 0.01), and no significant decline was observed in the experimental group. The hemagglutination scores of Fy^b^, Jk^b^, and s were sustained at the same level in the experimental group during 70-day storage.

**Figure 3.**
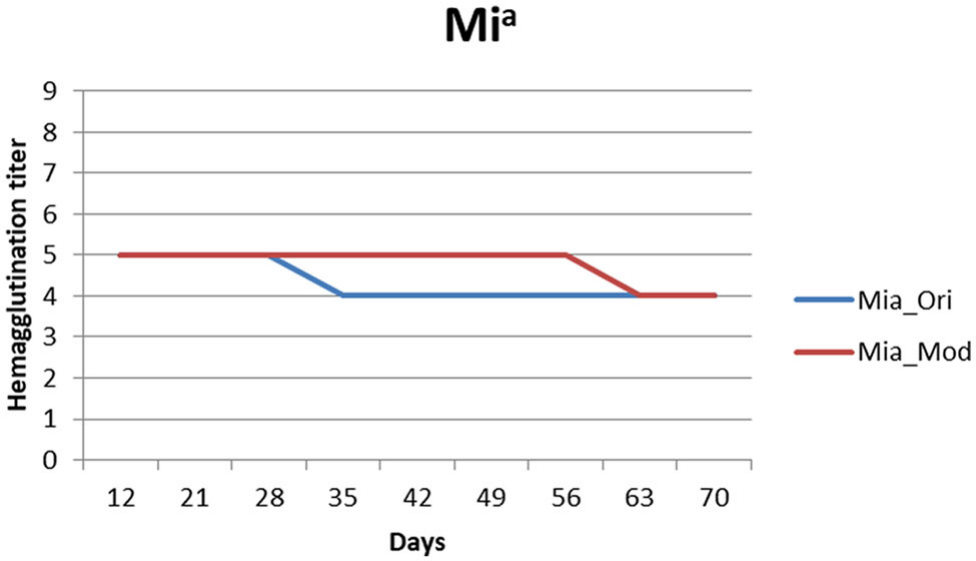
Mi^a^-positive screen cell antigenicity changes. Mi^a^_Ori: Mia-positive screen cells preserved in the control solution, Mi^a^_Mod: Mi^a^-positive screen cells preserved in the experimental solution (the results were all quadruplicated which there were duplicate in two medical centers) The results presented in Figure 3 are means, and error bars (standard variation) are not shown because the repeated results were the same. The score in both experimental and control solutions decreased by 1 point, but the experimental solution had a higher score from day 35 to day 56.

The Mi^a^ antigen reactivity remained detectable throughout the storage period in both groups (Figure 3), and the hemagglutination reactivity was only 20% lower on day 70. The experimental solution, however, preserved the original hemagglutination activity (score: 5) of the Mi^a^ antigen longer than the control solution (56 days versus 28 days).

## Discussion

Positive hemagglutination in pretransfusion testing indicates the presence of RBC antibodies in the serum. However, hemagglutination activity in commercial RBC reagents usually expresses a time-dependent decline and probably causes a false negative reaction for allo-antibody detection, especially when expiring or outdate RBC reagents are used. In addition, early haemolysis of RBC reagents can result in false-positive reactions and decrease the sensitivity of pretransfusion antibody screening and identification assays. Therefore, the development of a new RBC storage solution to maintain RBC integrity is crucial for accurate pretransfusion testing. Compared with the conventional storage solution, the decline of RBC hemagglutination scores in the new solution was often later than that in the control solution for most antigen phenotyping, especially for labile antigens such as D, P1, and M. Moreover, our data indicated that RBCs stored in the new solution exhibited delayed haemolysis up to 70 days storage.

Generally, earlier drops of hemagglutination reactivity in the control solution were noted for almost all antigens in SI and SII cell panels. The original hemagglutination reactivity of D, Jk^b^, M, C, e, and P1 antigens could only be preserved for 21 days. In our experiment, the hemagglutination reactivity of the Rh system antigens (D, C, E, c, and e) declined by approximately 25%–50% during the original storage period. Approximate to the 40% drop reported in previous study [14].

The sharp decline in the ‘M’ titre score between day 63 and day 70 is the actual result we obtained from the experiment, which indicated the uppermost storage limit of the ‘M’ antigen is 63 days in the conventional RBC solution. Although the cause of ‘s’ titre differences between two RBC panel cells needs further investigation, we suspect that it is due to different antigen degradation occurring in different donors (SI and SII).

The experimental group exhibited an extended shelf life of RBCs up to 70 days with a smaller antigenicity decline than the control solution. The P1 antigen demonstrated the highest decline in hemagglutination reactivity during storage, consistent with previous reports [12,14]. However, the hemagglutination reactivity of the P1 was maintained in the experimental group on day 70. Although studies have reported that the Lewis antigen is the most labile during storage [12], only a moderate loss of Lewis antigenicity was observed in the present study. Different storage solutions might have distinct preservation capabilities for different antigens.

The experimental solution provided superior protection against haemolysis and preserved most original hemagglutination reactivities of RBC antigens, including labile antigens (D, P1, and M) and the domestic Mi^a^ antigen, over the 70-day storage period.

Some studies have suggested that the addition of PEG leads to the loss of antigenicity in human RBCs, and PEG has been used to produce universal/stealth RBCs to reduce certain antigenicities [23]. However, attenuated RBC antigenicity was not observed in our experiments. Nevertheless, the value of using PEG in the indirect antiglobulin test on RBCs has been verified [24]. The preservation of RBC antigenicity was enhanced with the addition of PEG in the modified Alsever’s solution, and we suspect that the concentration and molecular weight of PEG and other components of the solution may have caused this improvement in RBC antigenicity. The effects of PEG on RBCs warrant more scholarly attention.

Our study has some limitations. First, our results were only conducted using only Sanquin’s commercial and Formosa Biomedical Technology Corporation RBCs. Future studies should include different RBCs and antibody sources to validate the generalization of preservation efficacy. Second, few studies have reported the development of a novel RBC solution for extending the shelf life of reagent RBCs, particularly focusing on maintaining RBC antigenicity; accordingly, the relevant references may be somewhat old. Once the research involved in innovation-level investigation worth patenting, the researchers would not publish the data before patent licensing. Owing to copyright protection and intellectual property rights, the exact formula of the novel solution cannot be disclosed [25]. Nonetheless, any innovative reagent with good performance should generate reproducible results regardless of whether the work is published. Metek Lab Company can provide the solution to any researchers interested in replicating our study.

In conclusion, our data indicate that the new RBC storage solution containing PEG can provide superior protection against haemolysis and preserve most of the hemagglutination reactivity of RBC antigens, including labile antigens (D, P1 and M) and domestic Mi^a^ antigens during the 70-day storage period. The new RBC storage solution may therefore be a better choice to reduce reagent cost, which requires monthly renewal of reagent lot. However, future studies are warranted to elucidate the RBC preservation and antigen preservation mechanisms of the new RBC storage solution. On the basis of our result, antigenicity scores for detecting antigenicity preservation should be at least higher than 5 points. This threshold could be applied to the development of innovative reagents in the future.

## Data Availability

The original contributions presented in the study are included in the article. Further inquiries can be directed to the corresponding author.
